# Effect of Essential Oil of Thyme (*Thymus vulgaris* L.) or Increasing Levels of a Commercial Prebiotic (TechnoMOS^®^) on Growth Performance and Carcass Characteristics of Male Broilers

**DOI:** 10.3390/ani11113330

**Published:** 2021-11-22

**Authors:** Hossein Amouei, Giulia Ferronato, Ali Ahmad Alaw Qotbi, Mehrdad Bouyeh, Peter G. Dunne, Aldo Prandini, Alireza Seidavi

**Affiliations:** 1 Department of Animal Science, Rasht Branch, Islamic Azad University, Rasht 41335-3516, Iran; hossein_amouei@yahoo.com (H.A.); adian2000@yahoo.com (A.A.A.Q.); booyeh@iaurasht.ac.ir (M.B.); 2 Department of Animal Sciences, Food and Nutrition (DIANA), Università Cattolica Sacro Cuore, 29122 Piacenza, Italy; giulia.ferronato@unicatt.it (G.F.); aldo.prandini@unicatt.it (A.P.); 3 Department of Applied Sciences, Dundalk Institute of Technology, Dublin Road, A91 K584 Dundalk, Ireland; peterdunne34@gmail.com

**Keywords:** *Thymus vulgaris*, prebiotics, feed conversion ratio, growth, broiler

## Abstract

**Simple Summary:**

Since the ban of in-feed antimicrobial with auxin purposes, a wide range of herbal plants have been tested as growth promoters. In particular, thyme and prebiotic compounds, such as mannan-oligosaccharides, have been tested in animal production due to their antibacterial, antifungal, and antioxidant properties and antimicrobial, anticoccidial, and anti-inflammatory actions. Thyme essential oils and TechoMOS^®^ positively affected the growth performance of Ross male broiler during the grower phase, reducing the feed intake and improving feed conversion ratio. However, increasing dose of TechoMOS^®^ only affected the broiler performance on a week-to-week basis and no difference has been registered between same dosage of TechoMOS^®^ and TEO. Dietary treatments, both TechoMOS^®^ and TEO, did not affect the carcass characteristics at 42 days.

**Abstract:**

To investigate the effect of thyme (*Thymus vulgaris* L.) essential oil (TEO) or increasing inclusion of a prebiotic (TechnoMOS^®^) on growth performance and carcass characteristics of Ross 308 broilers, 400 one-day-old male broilers (43.5 g, as mean of body weight) were placed in 20 pens (2.0 × 1.0 m, with a floor area of 0.10 m^2^ per bird) in groups of 20, and each pen cage was assigned to a specific dietary treatment (four replicates per each one). The dietary treatments included basic diet (no additive; CTR), basic diet including 0.025%, 0.075%, or 0.125% of TechnoMOS^®^ (MOS025, MOS075, and MOS125, respectively), or basic diet including 0.075% thyme extract (TEO075). All dietary treatments were offered from the beginning of the study until the end of the trial. There were no effects of MOS or TEO on carcass characteristics. No significant effects of treatment on weight gain were obtained on a week-by-week basis; however, CTR birds gained less weight during the grower phase and overall compared with MOS birds. The same contrast for feed intake revealed that CTR birds had greater feed intake than MOS birds during both the grower phase and overall (492.18 g and 486.35 g, respectively). In conclusion, treated groups showed an improved feed conversion ratio.

## 1. Introduction

The poultry sector is considered as the fastest growing and most flexible of all the livestock sectors [1] and is frequently subjected to various kind of stresses due to intensive production pressure at farming level [2]. According to the increase of consumer’s demand of poultry products, the agricultural stage is faced with the need to improve the animal welfare conditions [3] and to reduce the environmental impact of the production cycle. Not least, in recent decades particular attention has been focused on reducing the use of antibiotics [4], through the reduction of disease incidence, better feed efficiency, and feed conversion ratio. Optimizing broiler health, and in particular gastrointestinal health, is a critical aspect necessary to achieve optimum performance in the relatively short production cycle.

In-feed antimicrobials have been used for decades, generating further challenges such as the antibiotic-resistance in the human and domestic livestock and poultry microbiota, reflecting what Aminov [5] referred to as the “interminable adaptive capabilities of bacteria”.

The trend towards decreased use of historical antibiotic compounds in animal production, for prophylactic and growth-promotion purposes, was codified at European level in 2006 [6] leading to the adoption of a wide range of herbal plants, their extracts, essential oils, and purified components as replacements [7]. Moreover, a broad range of substances, including organic acids [8], mannan- and fructo-oligosaccharides, prebiotics [9], bacterial probiotics [10], enzymes [11,12,13], and synbiotics [14,15,16] have been tested under experimental and commercial conditions.

Thyme (*Thymus vulgaris* L.), an aromatic member of the *Lamiaceae* family, is a recognized source of essential oil with several food, feed, and medicinal applications. Indeed, in vitro experiments showed pharmacological activities [17]. Different plant species could be different in content and type of components [18]; however, thyme generally contains essentials oil, carvacrol, flavonoids, and phenol compounds [19,20]. Regarding the thyme essential oil, this mainly contains oxygenated monoterpenes, monoterpene hydrocarbons, sesquiterpene hydrocarbons and oxygenated sesquiterpenes like thymol (10–64%) [11,21], as well as carvacrol (2–11%), γ-terpinene (2–31%) and p-cymene (10–56%) [11,22]. Thyme is also a source of several vitamins such as B-complex, folic acid, beta-carotene, vitamin A, K, E, and C, and minerals like potassium, calcium, iron, manganese, magnesium, and selenium [23].

Taken together, the composition of thyme essential oil leads to antiseptic, antibacterial, antifungal, and antioxidant properties and antimicrobial, anticoccidial, and anti-inflammatory actions [23,24]. Thyme essential oil has been shown to increase the production of digestive enzymes, which in turn improve the digestion of nutrients [25].

Another important feed additive is represented by prebiotic compounds. These substances are non-digestible small carbohydrate fragments, called oligosaccharides, and are composed of galactose, fructose, or mannose monosaccharides [26,27]. Prebiotics have gained increased attention as beneficial feed additives and have been reported to have a positive effect at gastrointestinal level mainly stimulating the growth of positive bacteria and reducing pathogenic bacteria [26,28,29,30,31,32]. Among prebiotic substances, mannan-oligosaccharides (MOS) have been extensively studied as an alternative to antibiotic growth promoters in broiler diets. In this regard, MOS are perceived as more ‘natural’, feasible, and affordable alternatives to traditional antibiotic growth promoters which do not incur the disadvantages of microbial resistance or contaminating residues [33]. MOS are typically derived from yeast outer cell walls which consist of proteins and carbohydrates including glucose, mannose, and N-acetylglucosamine. Briefly, the way of action of MOS involves provision of alternative attachment sites on MOS for pathogenic bacteria which prevents or reduces bacterial adhesion to intestinal enterocytes and the adhesion-induced pathogenesis [34,35,36,37,38]. Other mechanisms may include effects on villus height (uniformity and integrity) and immune system stimulation [39,40,41,42,43,44]. Additionally, MOS have been shown to effect gene expression in the jejunum which could contribute to their observed beneficial effects [45,46,47].

The objective of the present study was to investigate the effects of thyme extract and of three levels of a commercial prebiotic product (TechnoMOS^®^) on growth performance and carcass characteristics of male broiler chicks.

## 2. Materials and Methods

### 2.1. Animals, Housing, and Experimental Design

Four hundred one-day-old Ross 308 male broiler chickens (Aviagen, Newbridge, Scotland, UK) purchased from a commercial hatchery were involved in a 42-days study.

The birds were randomly allocated into 5 treatment groups, composed by 2 birds, with 4 replicates per each treatment. The broiler chickens were placed in 20 cages (2.0 × 1.0 m) with earth flooring (floor area of 0.10 m^2^ per bird covered with paper roll litter) in a thermostatically controlled curtain side-wall poultry barn for the entire duration of the experiment.

The farming conditions were set according to the standard brooding practice for the rearing stages of the birds, as reported in *Ross Broiler Management Manual* [48].

Relative humidity was maintained between 55–65%. The initial temperature was kept at 32 °C, was decreased periodically to 24 °C at 3 weeks of age, and maintained until the end of the study. Constant light (23 W fluorescent tubes in ceiling fixtures) was provided on day 1, and then it was established at 23 h per day until the end of the study. Air circulation within the poultry barn was provided by tunnel ventilation.

### 2.2. Experimental Diets

A two-phase feeding program, according to Ross 308 catalogue recommendations (Aviagen, 2014), was used and consisted of provision of starter feed from the 1 to the 21 days of age, and of grower feed from the 22 to the 42 days of age (Table 1 and Table 2) [49]).

The dietary treatments used were as follows: (1) basic diet (no additive; CTR); (2) basic diet including 0.025% (0.25 g/kg) TechnoMOS^®^ (MOS025); (3) basic diet including 0.075% (0.75 g/kg) TechnoMOS^®^ (MOS075); (4) basic diet including 0.125% (1.25 g/kg) TechnoMOS^®^ (MOS125); (5) basic diet including 0.075% (0.75 g/kg) thyme extract (TEO075). All dietary treatments were offered from the beginning until the end of the trial.

The additives used in the current experiment were a commercial prebiotic product, TechnoMOS^®^ (Biochem, Kuestermeyerstraße 16, D-49393, Lohne, Germany), and thyme essential oil extract product (Zarband Co., Tehran, Iran).

### 2.3. Growth Performance, Feed Intake and Carcass Characteristics

Body weight (BW) and feed intake was weekly recorded throughout the study for each replicate within each treatment. Average of feed intake, average weight gain, and feed conversion ratio (FCR) was also calculated.

At slaughter (42 d of age), 4 birds per replicate (16 birds per treatment) were randomly selected, weighed, and killed by cervical dislocation by qualified personnel. After slaughter and picking operations, the head and legs were removed. Broilers were eviscerated before determining the carcass components. Weights of defeathered body weight, breast, thighs, wings, abdominal fat, vertebrae, gastrointestinal (GIT), liver, heart, pancreas, spleen, and gizzard were also assessed. Furthermore, the eviscerated carcass weight was measured.

### 2.4. Statistical Analysis

Data were analyzed by SAS (version 9.2; Institute Inc., Cary, NC, USA) using the generalized linear model (GLM) procedure (completely randomized design with five treatments and four replicates per treatment). The post-hoc analysis was performed by Duncas test. Linear (L) and quadratic (Q) effects of increasing MOS inclusion levels were estimated. Contrasts were performed between CTR and MOS, CTR and TEO075, and between TEO075 and MOS075 to assess differences between CTR and MOS, between CTR and thyme, and between MOS and thyme inclusion at 0.75 g/kg, respectively. The significance was declared at *p* ≤ 0.05.

## 3. Results

### 3.1. Growth Performance

Overall and weekly weight gains are reported in Table 3.

Diet treatments affect weight gain (*p *=** 0.043) during the first (1 to 7 days) and the last (36 to 42 days) week of the trial. Specifically, dietary treatments showed a significant effect during the grower phase (22 to 42 days) of broilers. During the first week, weekly weight gains of CTR broilers resulted the greatest (113.73 ± 2.13 g), whereas for TEO075 group the lowest (107.6 ± 2.82 g; *p *=** 0.043). In the last week, CTR group had the lowest value of weight gain (543.50 ± 121.83 g) and MOS125 the greatest (869.75 ± 59.42 g; *p *=** 0.040). Moreover, no significant differences have been found between MOS groups, MOS025 or MOS075, and TEO075 group. During the middle weeks of the trial, overall MOS or TEO treatments did not show effects on weight gains. Treatments affected weekly weight gain only during the grower phase (*p *=** 0.047). CTR group showed the lowest weight gain (1750.85 ± 101.46 g) compared with the others treatment groups. Specifically, the treatment MOS125 led to the greatest weight gain (2187.37 ± 67.77 g).

The level of treatment showed a linear effect during the grower phase (22 to 42 days), during the week 36 to 42 (*p *=** 0.014), and the overall period (1 to 42 days) (*p *=** 0.027 and *p* = 0.045, respectively). A quadratic effect was registered only during the first week of trial (*p* = 0.03). Hence, the weight gain values of MOS and TEO075 groups resulted overall lower than CTR group in the period 1 to 7 days (*p *=** 0.0086 and *p *=** 0.0057, respectively) and 36 to 42 days (*p *=** 0.0064 and *p *=** 0.0473 respectively); likewise, a similar response was reached during the grower phase and the overall cycle period.

In addition, no differences were found between MOS075 and TEO075 for weekly weight gain or mean weight gains during the three phases of production.

### 3.2. Weekly Feed Intake

Feed intake data are reported in Table 4.

Average feed intake was significantly greater (*p* < 0.001) for CTR (141.50 ± 2.38 g) than for all other treatments from day 1 to 7 days.

By the second week (8 to 14 days), CTR birds had the lowest feed intake (300.75 ± 6.99 g; *p* < 0.001) compared to other treatment groups.

There were weekly differences in feed intake between treatments in the fourth week (*p *=** 0.024), fifth week (*p *=** 0.052), and sixth week (*p *=** 0.003). In the fourth week, feed intake for MOS125 group was significantly (*p *=** 0.052) greater (937.36 ± 38.33 g) than MOS025 and MOS075 and TEO075, whereas feed intake of MOS025 and TEO075 was significantly (*p* = 0.052) lower (787.35 ± 86.45 g and 797.40 ±89.79 g) compared with CTR and MOS125 (922.50 ± 89.95 g and 937.36 ± 38.33 g, respectively). MOS treatment, both at 0.75 g/kg and 0.125 g/kg dose, was different from MOS025 and TEO075.

During the fifth and sixth weeks, weekly feed intake of CTR group was the greatest (1221.25 ± 78.78 g and 1506.25 ± 67.75 g, respectively), whereas for MOS025 group was the lowest (953.99 ± 176.06 g and 1130.75 ± 147.51 g, respectively) (*p *=** 0.052). The greater feed intake of CTR broilers was achieved in the grower phase (*p *=** 0.011), where it was greater (*p *=** 0.011) than MOS025 but not different to MOS075, MOS125, or TEO075. Similarly, overall feed intake for CTR broilers was significantly (*p *=** 0.011) greater than MOS025, but was not significantly different to MOS075, MOS125, or TEO075.

There were linear effects of increasing MOS inclusion in the first (*p* = 0.002) and second (*p* < 0.001) week. A quadratic effect of increasing MOS inclusion was observed in the third week (*p* = 0.011), fourth week (*p* = 0.006), and sixth week (*p *=** 0.003). Increasing MOS inclusion had a quadratic effect on feed intake in the grower phase (*p *=** 0.007) and overall (*p* = 0.005).

When contrasts were performed, it was observed that feed intake for CTR broilers was significantly greater than MOS broilers in the first (*p *=** 0.0001) and last (*p *=** 0.0005) week of the production cycle. Conversely, feed intake of CTR was lower (*p* < *0*.001) than MOS in the second week. Feed intake of CTR was greater (*p *=** 0.011) than MOS in the grower phase and overall (*p *=** 0.007 and *p *=** 0.009, respectively).

### 3.3. Weekly Feed Conversion

Feed conversion ratio (FCR) data are reported in Table 5.

There were differences in FCR between treatments in the second (*p *=** 0.033), fifth (*p *=** 0.029), and sixth week (*p *=** 0.005). In the second week, CTR value was significantly lower than MOS025 and MOS125 (1.20± 0.06) (*p *=** 0.033). In the fifth week, CTR group was significantly higher than MOS025, which in turn was found to be the lowest (1.64 ± 0.07 g and 1.36 ± 0.12 g). During the sixth week, the CTR group (1.89 ± 0.04 g) was found to be significantly higher than MOS025, MOS075, and TEO075 (1.38 ± 0.14 g, 1.54 ± 0.21 g and 1.47 ± 0.24 g, respectively). Furthermore, the CTR group showed greater FCR during both the grower phase (2.09 ± 0.09, *p *=** 0.002) and overall period (1.89 ± 0.04, *p *=** 0.005) compared with MOS and TEO treatments.

There were linear effects of dietary treatments inclusion during the first, second, fourth, and sixth week (*p *=** 0.028, 0.026, 0.036, and 0.043) as well as during the grower phase and overall (*p *=** 0.017 and 0.043). Quadratic effect of treatments inclusion was observed in the fourth, fifth and sixth week (*p *=** 0.010, 0.003, and 0.006) and during the grower phase and overall (*p *=** 0.006).

Contrast analysis revealed a treatment effect both for the MOS and TEO075 during the first (*p *=** 0.0355 and *p *=** 0.0372, respectively), second (*p* = 0.0026 and *p *=** 0.0339, respectively), fifth (*p *=** 0.0243 and *p *=** 0.0184, respectively), and sixth week (*p *=** 0.0006 and *p *=** 0.0010, respectively).

### 3.4. Carcass Characteristics

Carcass characteristics are presented in Table 6.

There was no significant effect of treatment on any carcass characteristics. There was only a significant quadratic effect (*p *=** 0.021) of MOS inclusion on thigh weight.

## 4. Discussion

Several studies investigated and reported thyme supplementation effects on blood parameters, growth performance, gut integrity and content, and carcass characteristics of broilers [49,50,51,52,53,54,55,56,57,58,59,60]. Specifically TechnoMOS^®^ was also tested, either by using it alone or symbiotically with probiotics, and confirmed a positive effect on performance (i.e., weight gain, FCR) [61,62,63]. Abudabos et al. and Murshed et al. [61,62], unlike our study, used different doses according to the cycle phase and obtained better performance testing MOS alone compared to its combination with probiotics. It is known that animal requirements and digestive capacity, according to digestive enzyme production and intestinal mucosa development, varies according to the animal age [64,65]. These could influence the effective required dose. Moreover, farm management, feeding strategies, and pen hygiene could also affect the animal performance. These factors may modify the impact of MOS dietary inclusion.

From the present study, it could be pointed out that feed additive inclusion may change slightly during the production cycle. However, the latter can only be assessed when parameters are measured weekly. During the first week of treatment, no differences were detected for FCR values, even though weekly feed intake and weight gain were affected. This could be explained by the small quantity of feed consumed during this period. In this study the overall effect of treatments mainly occurred during the grower phase and overall period, and this was in line with previous studies.

Sojoudi et al. [66] used an increasing level of MOS supplementation (TechnoMOS^®^) in the diet of Ross 308 broilers. Authors reported that total feed intake, total consumed energy (kcal), and total consumed protein were significantly higher than control group when MOS was included in the basic diet at the lowest inclusion of 0.1% or at 0.15%. Thus, since inclusion of MOS likely represents a significant component of the cost of feed, if no additional benefits or efficiencies can be gained by including levels above 0.1%, it does not make economic sense to constrain inclusion to that level.

In a meta-analysis of broiler chicken pen trials evaluating MOS over a decade (1993–2003), Hooge [39] reported that MOS has a positive effect, improving average body weight and reducing FCR and mortality incidence; moreover, authors suggested that the optimal recommended MOS inclusion level in broiler diets is 0.2% between 0 and 7 days, 0.1% between 7 and 21 days, and 0.05% between 21 and 42 days, implying that scope exists to calibrate inclusion levels, a rationale based on a number of factors. In the present study, the inclusion levels used were similar, even though lower (for 0.025% and 0.075%) than those used by Sojoudi et al. [67], and not different from those recommended by Hooge [39]. The highest inclusion level, 0.125%, was slightly higher than the level used by Sojoudi et al. [67].

Our findings regarding TEO effect showed more effectiveness than MOS treatment at the same dosage. However, MOS also showed a significant effect on weekly feed intake during the second and third weeks. Previous studies confirmed that TEO had positive effect on immune functions [54,68], FCR, and growth [56,69]. In our study TEO was fed only at 0.75 g/kg dose, but Attia et al. and Wade et al. [56,69] suggested that the optimal dose should be set at 1 g/kg; Zhu et al. [68] in contrast, found the optimal dose range to be 0.1 to 0.25 g/kg.

In the present study, significant effects of treatment on weight gain were largely absent on a week-by-week basis, but a contrast between CTR and MOS for weight gain revealed that CTR broilers gained less weight than MOS during the grower phase and overall. The same contrast for feed intake revealed that CTR broilers had greater feed intake than MOS broilers during both the grower phase and overall. Thus, it can be speculated that MOS supplementation led to a better feed efficiency, indicating a beneficial effect of MOS compared with CTR. A similar trend was also observed for TEO, also implying a positive effect of this feed supplement for feed efficiency. Even though a cost-benefit analysis is beyond the aim of the present study, clearly an economic assessment of MOS and TEO use can be faced and taken into account with local availability and the price of prebiotic or bioactive feed additives.

It is widely known that physiological effects of MOS and TEO are mediated by their effects on the chicken intestinal microbiota. The interaction of the gut microbiota with host physiology, both locally in the gut and in more systemic ways, is now emerging as a core concern in livestock production [70,71]. It is also noteworthy that the chicken gut microbiota changes during the production cycle [72]; indeed, the microbiota during early production reflect the maternal influence [73,74] and subsequent changes reflect diet and environment [75]. Thus, if the purpose of supplementing with prebiotic feed additives is to modify performance and health parameters, the use of these compounds should take into account the microbial community dynamics with the aim of being able to have a greater effect. For instance, the fact that MOS broilers gained 376.58 g more weight than CTR broilers during the grower phase (between days 22 and 42) and only 353.83 g more weight than the CTR broilers over the entire production cycle suggests that beneficial effects of MOS supplementation are time dependent. Temporal variation has been observed in the chicken intestinal microbiome [76] and, thus, mediation of such time-dependent effects of prebiotic feed additives on the physiology of the host broilers could be speculated from the present study.

Kakebaveh et al. [77] showed that prebiotics and probiotics promoted the Lactobacilli population in the intestinal broiler microbiota, and also efficacy on challenged broiler with *Salmonella* spp. or *Clostridium perfringens*, promoting the surface area of intestinal villi and immune parameters [78,79].

Although there were positive effects on production performance in the present study, there were no effects on carcass characteristics measured, which was in line with data reported by Salehimanesh et al. and De Souza et al. [80,81]. On the contrary, Tayeri et al. [82] showed effects on carcass composition with symbiotic use of probiotics and prebiotics and Ahmadian et al. [83] using thyme essential oil.

## 5. Conclusions

In conclusion, taking together all the results, the tested prebiotic at different dosage and thyme essential oil affected the grower and overall growth performance of male Ross broiler during a 42-day trial. Considering that the dosage was not modified during the entire trial, the weekly response was different and with a marked action during the grower phase. The latter suggests that the effectiveness could be linked with the feed and feed additive intake. The main effect, of both MOS and TEO, revealed better weight gain, feed intake, and feed conversion ratio but no improvements on carcass traits compared with control group.

Even though average weight gain and feed intake have been influenced by treatments, no differences in feed conversion ratio had been found between treatments. Moreover, at similar dose level, MOS and TEO treatments did not show differences. Probably a different dosage should be used according mainly to the week of life, in order to reach a greater effect from a performance but also economic standpoint.

## Figures and Tables

**Table 1:** Ingredient composition of diets during the starter (1–21 days of age) and grower (22–42 days of age) periods.

Ingredient (%, Fresh Weight Basis)	Starter Period (1–21 Days of Age)	Finisher Period (22–42 Days of Age)
Corn	54.32	58.69
Soybean Meal	39.43	31.87
Corn oil	2.16	5.83
Mineral oysters	0.90	0.79
Dicalcium phosphate dihydrate	2.05	1.68
NaCl	0.37	0.37
DL-Methionine	0.20	0.22
Lysine-HCl	0.07	0.05
Vitamin and mineral premix ^#^	0.50	0.50
Total	100	100

^#^ Supplied per kilogram of feed: vitamin A, 12,500 I.U.; vitamin D3, 1250IU; vitamin E 18IU; vitamin K3 3.7 mg; thiamine 1.8 mg; riboflavin 6.6 mg; calcium pantothenate 10 mg; niacin, 37.5 mg; pyridoxine, 32.5 mg; vitamin B12 2.5 mg; manganese 50 mg; zinc, 37.5 mg; iron, 25 mg, copper 7.5 mg.

**Table 2:** Proximate composition and specific nutrient analysis of diets during the starter (1–21 days of age), and grower (22–42 days of age) periods.

Nutrient Analysis ^#^	Starter Period (1–21 Days of Age)	Finisher Period (22–42 Days of Age)
Metabolizable Energy (ME, kcal/kg)	2900	3200
Metabolizable Energy (ME, MJ/kg)	12.13	13.38
Crude protein (%)	22.16	19.20
Calcium (%)	1.00	0.85
Available Phosphorus (%)	0.50	0.42
DCAB (mEq/kg) ^§^	236	202
Lysine (%)	1.15	0.96
Methionine (%)	0.50	0.48
Methionine + Cysteine (%)	0.83	0.78
Threonine (%)	0.79	0.71

^#^ Calculated nutrients (according to the National Research Council, 1994). ^§^ DCAB: dietary cation-anion balance, expressed as milliequivalents per kg.

**Table 3:** Mean weekly weight gain (g) and mean weight gains (g) during the starter (1 to 21 days), grower (22 to 42 days), and overall (1 to 42 days) production periods for Ross 308 broilers offered increasing levels of a commercial prebiotic (TechnoMOS).

	Dietary Treatments ^#^							Effects		Contrasts		
Days	CTR	MOS025	MOS075	MOS125	TEO075	SD	*p* Value	L	Q	CTR v. MOS	CTR v. TEO075	MOS075 v. TEO075
1–7	113.73 ^b^	108.97 ^ab^	108.25 ^ab^	109.88 ^ab^	107.60 ^a^	1.90	0.043	0.112	0.030	0.0086	0.0057	0.7373
8–14	212.12	208.19	222.06	210.21	219.57	8.42	0.421	0.769	0.311	0.8446	0.3900	0.7710
15–21	381.30	361.92	374.91	348.80	380.76	21.24	0.507	0.291	0.754	0.2803	0.9800	0.7863
22–28	539.35	548.93	518.78	523.87	500.07	45.60	0.843	0.610	0.908	0.8156	0.4023	0.6873
29–35	668.00	803.25	832.50	793.75	824.75	84.80	0.334	0.230	0.165	0.0585	0.0845	0.9284
36–42	543.50 ^a^	785.00 ^ab^	706.50 ^ab^	869.75 ^b^	747.00 ^ab^	94.20	0.040	0.014	0.684	0.0064	0.0473	0.6733
Starter	707.15	679.08	705.23	668.88	707.93	20.79	0.236	0.262	0.599	0.2000	0.9704	0.8982
Grower	1750.85 ^a^	2137.18 ^ab^	2057.78 ^ab^	2187.37 ^b^	2071.82 ^ab^	136.60	0.047	0.027	0.265	0.0042	0.0329	0.9195
Overall	2458.00	2816.25	2763.00	2856.25	2779.75	143.10	0.090	0.045	0.252	0.0085	0.0400	0.9084

^#^ CTR: basic diet (control, no additive); MOS025: basic diet including 0.025% (0.25 g/kg) TechnoMOS^®^; MOS075: basic diet including 0.075% (0.75 g/kg) TechnoMOS^®^; MOS125: basic diet including 0.125% (1.25 g/kg) TechnoMOS^®^; TEO075: basic diet including 0.075% (0.75 g/kg) thyme extract (Zarband Co., Tehran, Iran). SD: Standard Deviation. ^a,b^ Different letters represent significant differences among challenge treatments (*p* < 0.05).

**Table 4:** Mean weekly feed intake (g) and mean feed intakes (g) during the starter (1 to 21 days), grower (22 to 42 days), and overall (1 to 42 days) production periods for Ross 308 broilers offered increasing levels of a commercial prebiotic (TechnoMOS^®^) or thyme essential oil.

	Dietary Treatments ^#^							Effects		Contrasts		
Days	CTR	MOS025	MOS075	MOS125	TEO075	SD	*p* Value	L	Q	CTR v. MOS	CTR v. TEO075	MOS075 v. TEO075
1–7	141.50 ^b^	133.00 ^a^	132.50 ^a^	130.75 ^a^	129.75 ^a^	2.28	<0.001	0.002	0.058	0.0001	0.0001	0.2458
8–14	300.75 ^a^	349.25 ^b^	355.25 ^b^	356.25 ^b^	352.00 ^b^	7.47	<0.001	<0.001	<0.001	<0.0001	<0.0001	0.6696
15–21	630.00	585.75	582.44	609.04	572.44	22.97	0.138	0.411	0.011	0.0634	0.0248	0.6695
22–28	922.50 ^bc^	787.35 ^a^	819.80 ^ab^	937.36 ^c^	797.40 ^a^	51.50	0.024	0.329	0.006	0.0975	0.0282	0.6698
29–35	1221.25 ^b^	953.99 ^a^	1160.78 ^ab^	1168.31 ^ab^	1064.41 ^ab^	86.10	0.052	0.513	0.124	0.0910	0.0884	0.2804
36–42	1506.25 ^b^	1130.75 ^a^	1233.30 ^a^	1281.84 ^ab^	1201.16 ^a^	80.30	0.003	0.097	0.003	0.0005	0.0017	0.6945
Starter	1072.25	1068.00.0	1070.19	1096.04	1054.19	28.12	0.683	0.277	0.407	0.8032	0.5304	0.5778
Grower	3650.00 ^b^	2872.09 ^a^	3213.88 ^ab^	3387.51 ^ab^	3062.98 ^ab^	193.40	0.011	0.873	0.007	0.0071	0.0083	0.4473
Overall	4722.25 ^b^	3940.09 ^a^	4284.06 ^ab^	4483.55 ^ab^	4117.16 ^ab^	198.70	0.011	0.987	0.005	0.0090	0.0082	0.4141

^#^ CTR: basic diet (control, no additive); MOS025: basic diet including 0.025% (0.25 g/kg) TechnoMOS^®^; MOS075: basic diet including 0.075% (0.75 g/kg) TechnoMOS^®^; MOS125: basic diet including 0.125% (1.25 g/kg) TechnoMOS^®^; TEO075: basic diet including 0.075% (0.75 g/kg) thyme extract (Zarband Co., Tehran, Iran). SD: Standard Deviation. ^a–c^ Different letters represent significant differences among challenge treatments (*p* < 0.05).

**Table 5:** Feed conversion ratio (FCR, g/g), and mean FCR (g/g) during weeks 1 to 6 and the starter (1 to 21 days), grower (22 to 42 days), and overall (1 to 42 days) production periods for Ross 308 broilers offered increasing levels of a commercial prebiotic (TechnoMOS^®^) or thyme essential oil.

	Dietary Treatments ^#^							Effects		Contrasts		
Days	CTR	MOS025	MOS075	MOS125	TEO075	SD	*p* Value	L	Q	CTR v. MOS	CTR v. TEO075	MOS075 v. TEO075
1–7	0.90	0.87	0.87	0.852	0.86	0.02	0.135	0.028	0.769	0.0355	0.0372	0.4418
8–14	1.20 ^a^	1.34 ^b^	1.31 ^ab^	1.34 ^b^	1.30 ^ab^	0.04	0.033	0.026	0.148	0.0026	0.0339	0.9326
15–21	1.43	1.48	1.43	1.54	1.41	0.05	0.152	0.080	0.295	0.2366	0.6655	0.6486
22–28	1.55	1.46	1.49	1.65	1.48	0.07	0.096	0.036	0.010	0.7492	0.3348	0.9071
29–35	1.64 ^b^	1.36 ^a^	1.46 ^ab^	1.58 ^ab^	1.41 ^ab^	0.09	0.029	0.827	0.003	0.0243	0.0184	0.6090
36–42	1.89 ^b^	1.38 ^a^	1.54 ^a^	1.55 ^ab^	1.47 ^a^	0.11	0.005	0.043	0.006	0.0006	0.0020	0.5221
Starter	1.43	1.48	1.43	1.54	1.41	0.05	0.152	0.080	0.295	0.2366	0.6655	0.6486
Grower	2.09 ^b^	1.35 ^a^	1.59 ^a^	1.55 ^a^	1.50 ^a^	0.15	0.002	0.017	0.006	0.0002	0.0010	0.5275
Overall	1.89 ^b^	1.38 ^a^	1.54 ^a^	1.55 ^a^	1.47 ^a^	0.11	0.005	0.043	0.006	0.0006	0.0020	0.5221

^#^ CTR: basic diet (Control, no additive); MOS025: basic diet including 0.025% (0.25 g/kg) TechnoMOS^®^; MOS075: basic diet including 0.075% (0.75 g/kg) TechnoMOS^®^; MOS125: basic diet including 0.125% (1.25 g/kg) TechnoMOS^®^; TEO075: basic diet including 0.075% (0.75 g/kg) thyme extract (Zarband Co., Tehran, Iran). SD: Standard Deviation. ^a,b^ Different letters represent significant differences among challenge treatments (*p* < 0.05).

**Table 6:** Carcass characteristics on the 42nd day of age of Ross 308 broilers offered increasing levels (0.25, 0.75, or 1.25 g/kg of diet) of a commercial prebiotic (TechnoMOS^®^) or thyme essential oil (0.75 g/kg diet).

	Dietary Treatments ^#^							Effects	
Variable	CTR	MOS025	MOS075	MOS125	TEO075	SD	*p* Value	L	Q
Live weight (g)	2662.50	2545.00	2552.50	2573.75	2722.50	104.2	0.385	0.541	0.389
Defeathered body weight (g)	2097.50	1965.5	2003.75	2031.25	2136.25	87.3	0.327	0.729	0.297
Eviscerated carcass (%)	61.82	60.09	60.56	61.47	60.44	1.143	0.534	0.939	0.185
Breast weight (g)	582.75	526.25	542.00	546.50	550.25	37.9	0.671	0.589	0.358
Thighs weight (g)	488.50	464.50	438.25	494.50	478.25	23.98	0.192	0.891	0.021
Wings weight (g)	153.50	150.50	156.25	150.00	150.00	8.30	0.922	0.875	0.675
Abdominal fat (g)	45.75	52.75	45.0.00	42.00	61.2	12.16	0.539	0.491	0.665
Vertebrae (g)	375.25	356.75	358.75	353.00	388.75	16.28	0.199	0.301	0.626
GIT weight (g)	380.75	338.75	345.75	353.50	369.25	24.12	0.430	0.511	0.245
Liver weight (g)	75.50	69.75	68.25	74.75	76.75	7.55	0.740	0.988	0.250
Heart weight (g)	16.50	15.50	15.25	16.00	16.75	1.565	0.852	0.803	0.425
Pancreas weight (g)	5.75	6.25	6.50	5.25	6.75	0.856	0.443	0.454	0.094
Spleen weight (g)	3.25	2.75	3.50	3.25	3.25	0.447	0.573	0.534	0.931
Gizzard weight (g)	69.25	68.75	73.00	74.50	74.00	5.91	0.792	0.273	0.995

^#^ CTR: basic diet (Control, no additive); MOS025: basic diet including 0.025% (0.25g/kg) TechnoMOS^®^; MOS075: basic diet including 0.075% (0.75g/kg) TechnoMOS^®^; MOS125: basic diet including 0.125% (1.25g/kg) TechnoMOS^®^; TEO075: basic diet including 0.075% (0.75g/kg) thyme extract (Zarband Co., Tehran, Iran). SD: Standard Deviation.

## References

[1] McLeod A., Thieme O., MacK S.D. (2009). Structural changes in the poultry sector: Will there be smallholder poultry development in 2030?. Worlds Poult. Sci. J..

[2] Kiczorowska B., Samolińska W., Al-Yasiry A.R.M., Kiczorowski P., Winiarska-Mieczan A. (2017). The natural feed additives as immunostimulants in monogastric animal nutrition—A review. Ann. Anim. Sci..

[3] (2019). Rowe; Dawkins; Gebhardt-Henrich A Systematic Review of Precision Livestock Farming in the Poultry Sector: Is Technology Focussed on Improving Bird Welfare?. Animals.

[4] Mottet A., Tempio G. (2017). Global poultry production: Current state and future outlook and challenges. Worlds Poult. Sci. J..

[5] Aminov R.I. (2010). A brief history of the antibiotic era: Lessons learned and challenges for the future. Front. Microbiol..

[6] European Parliament and of the Council (2003). Regulation No 1831/2003 of the European Parliament and of the Council of 22 September 2003 on additivies for use in animal nutrition. Off. J. Eur. Union.

[7] Franz C., Baser K.H.C., Windisch W. (2010). Essential oils and aromatic plants in animal feeding—A European perspective. A review. Flavour Fragr. J..

[8] Ferronato G., Prandini A. (2020). Dietary supplementation of inorganic, organic, and fatty acids in pig: A review. Animals.

[9] Tiwari U.P., Fleming S.A., Rasheed M.S.A., Jha R., Dilger R.N. (2020). The role of oligosaccharides and polysaccharides of xylan and mannan in gut health of monogastric animals. J. Nutr. Sci..

[10] Aziz Mousavi S.M.A., Hosseini H.M., Mirhosseini S.A. (2018). A review of dietary probiotics in poultry. J. Appl. Biotechnol. Rep..

[11] Steiner T. (2009). Phytogenics in Animal Nutrition: Natural Concepts to Optimize Gut Health and Performance.

[12] Windisch W., Rohrer E., Schedle K., Steiner T. (2012). Phytogenic Feed Additives to Young Piglets and Poultry: Mechanisms and Application. Phytogenics in Animal Nutrition.

[13] Woyengo T.A., Bogota K.J., Noll S.L., Wilson J. (2019). Enhancing nutrient utilization of broiler chickens through supplemental enzymes. Poult. Sci..

[14] Dibaji S.M., Seidavi A., Asadpour L., Da Silva F.M. (2014). Effect of a synbiotic on the intestinal microflora of chickens. J. Appl. Poult. Res..

[15] Mousavi S.M.A.A., Seidavi A., Dadashbeiki M., Kilonzo-Nthenge A., Nahashon S.N., Laudadio V., Tufarelli V. (2015). Einfluss eines synbiotikums (Biomin^®^IMBO) auf die mastleistung von broilern. Eur. Poult. Sci..

[16] Dev K., Mir N.A., Biswas A., Kannoujia J., Begum J., Kant R., Mandal A. (2020). Dietary synbiotic supplementation improves the growth performance, body antioxidant pool, serum biochemistry, meat quality, and lipid oxidative stability in broiler chickens. Anim. Nutr..

[17] Cosentino S., Tuberoso C.I.G., Pisano B., Satta M., Mascia V., Arzedi E., Palmas F. (1999). In-vitro antimicrobial activity and chemical composition of Sardinian Thymus essential oils. Lett. Appl. Microbiol..

[18] Rota M.C., Herrera A., Martínez R.M., Sotomayor J.A., Jordán M.J. (2008). Antimicrobial activity and chemical composition of Thymus vulgaris, Thymus zygis and Thymus hyemalis essential oils. Food Control.

[19] Hosseinzadeh S., Jafarikukhdan A., Hosseini A., Armand R. (2015). The Application of Medicinal Plants in Traditional and Modern Medicine: A Review of Thymus vulgaris. Int. J. Clin. Med..

[20] Bakkali F., Averbeck S., Averbeck D., Idaomar M. (2008). Biological effects of essential oils—A review. Food Chem. Toxicol..

[21] Moghtader M. (2013). In vitro antifungal effects of the essential oil of Mentha piperita L. and its comparison with synthetic menthol on Aspergillus niger. Afr. J. Plant Sci..

[22] Burt S. (2004). Essential oils: Their antibacterial properties and potential applications in foods—A review. Int. J. Food Microbiol..

[23] Dauqan E.M.A., Abdullah A. (2017). Medicinal and Functional Values of Thyme (*Thymus vulgaris* L.) Herb. J. Appl. Biol. Biotechnol..

[24] Saki A.A., Kalantar M., Khoramabadi V. (2014). Effects of Drinking Thyme Essence (*Thymus vulgaris* L.) on Growth Performance, Immune Response and Intestinal Selected Bacterial Population in Broiler Chickens Effects of Drinking Thyme Essence (*Thymus vulgaris* L.) on Growth. Poult. Sci. J..

[25] Lee K.W., Everts H., Kappert H.J., Frehner M., Losa R., Beynen A.C. (2003). Effects of dietary essential oil components on growth performance, digestive enzymes and lipid metabolism in female broiler chickens. Br. Poult. Sci..

[26] Ganguly S. (2013). Supplementation of prebiotics, probiotics and acids on immunity in poultry feed: A brief review. Worlds Poult. Sci. J..

[27] Gibson G.R., Roberfroid M.B. (1995). Dietary modulation of the human colonic microbiota: Introducing the concept of prebiotics. J. Nutr..

[28] Ferket P.R., Parks C.W., Grimes J.L. (2002). Benefits of Dietary Antibiotic and Mannanoligosaccharide Supplementation for Poultry.

[29] Mahdavi S., Zakeri A., Mehmannavaz Y., Nobakht A. (2013). Comparative study of probiotic, acidifier, antibiotic growth promoters and prebiotic on activity of humoral immune and performance parameters of broiler chickens. Iran. J. Appl. Anim. Sci..

[30] Al-Khalaifa H., Al-Nasser A., Al-Surayee T., Al-Kandari S., Al-Enzi N., Al-Sharrah T., Ragheb G., Al-Qalaf S., Mohammed A. (2019). Effect of dietary probiotics and prebiotics on the performance of broiler chickens. Poult. Sci..

[31] Biswas A., Mohan N., Raza M., Mir N.A., Mandal A. (2019). Production performance, immune response and blood biochemical parameters in broiler chickens fed diet incorporated with prebiotics. J. Anim. Physiol. Anim. Nutr..

[32] Froebel L.K., Jalukar S., Lavergne T.A., Lee J.T., Duong T. (2019). Administration of dietary prebiotics improves growth performance and reduces pathogen colonization in broiler chickens. Poult. Sci..

[33] Teng P.Y., Kim W.K. (2018). Review: Roles of prebiotics in intestinal ecosystem of broilers. Front. Vet. Sci..

[34] Yalçinkaya l., Güngör T., BAfiALAN M., Erdem E. (2008). Mannan Oligosaccharides (MOS) from Saccharomyces cerevisiae in Broilers: Effects on Performance and Blood Biochemistry*.

[35] Sohail M.U., Hume M.E., Byrd J.A., Nisbet D.J., Ijaz A., Sohail A., Shabbir M.Z., Rehman H. (2012). Effect of supplementation of prebiotic mannan-oligosaccharides and probiotic mixture on growth performance of broilers subjected to chronic heat stress. Poult. Sci..

[36] Tarabees R., Gafar K.M., EL-Sayed M.S., Shehata A.A., Ahmed M. (2019). Effects of Dietary Supplementation of Probiotic Mix and Prebiotic on Growth Performance, Cecal Microbiota Composition, and Protection Against Escherichia coli O78 in Broiler Chickens. Probiotics Antimicrob. Proteins.

[37] El-Shall N.A., Awad A.M., El-Hack M.E.A., Naiel M.A.E., Othman S.I., Allam A.A., Sedeik M.E. (2020). The simultaneous administration of a probiotic or prebiotic with live Salmonella vaccine improves growth performance and reduces fecal shedding of the bacterium in Salmonella-challenged broilers. Animals.

[38] Kridtayopas C., Rakangtong C., Bunchasak C., Loongyai W. (2019). Effect of prebiotic and synbiotic supplementation in diet on growth performance, small intestinal morphology, stress, and bacterial population under high stocking density condition of broiler chickens. Poult. Sci..

[39] Hooge D.M. (2004). Meta-analysis of broiler chicken pen trials evaluating dietary mannan oligosaccharide, 1993–2003. Int. J. Poult. Sci..

[40] Oliveira M.C., Rodrigues E.A., Marques R.H., Gravena R.A., Guandolini G.C., Moraes V.M.B. (2008). Performance and morphology of intestinal mucosa of broilers fed mannan-oligosaccharides and enzymes. Arq. Bras. Med. Vet. Zootec..

[41] Stefaniak T., Madej J.P., Graczyk S., Siwek M., Łukaszewicz E., Kowalczyk A., Sieńczyk M., Bednarczyk M. (2019). Selected prebiotics and synbiotics administered in ovo can modify innate immunity in chicken broilers. BMC Vet. Res..

[42] Stefaniak T., Madej J.P., Graczyk S., Siwek M., Łukaszewicz E., Kowalczyk A., Sieńczyk M., Maiorano G., Bednarczyk M. (2020). Impact of prebiotics and synbiotics administered in ovo on the immune response against experimental antigens in chicken broilers. Animals.

[43] Asem-Hiablie S., Battagliese T., Stackhouse-Lawson K.R., Alan Rotz C. (2019). A life cycle assessment of the environmental impacts of a beef system in the USA. Int. J. Life Cycle Assess..

[44] Craig A.D., Bedford M.R., Hastie P., Khattak F., Olukosi O.A. (2019). The effect of carbohydrases or prebiotic oligosaccharides on growth performance, nutrient utilisation and development of small intestine and immune organs in broilers fed nutrient-adequate diets based on either wheat or barley. J. Sci. Food Agric..

[45] Yang Y., Iji P.A., Choct M. (2007). Effects of Different Dietary Levels of Mannanoligosaccharide on Growth Performance and Gut Development of Broiler Chickens. Asian-Australas. J. Anim. Sci..

[46] Slawinska A., Dunislawska A., Plowiec A., Radomska M., Lachmanska J., Siwek M., Tavaniello S., Maiorano G. (2019). Modulation of microbial communities and mucosal gene expression in chicken intestines after galactooligosaccharides delivery In Ovo. PLoS ONE.

[47] Soumeh E.A., Mohebodini H., Toghyani M., Shabani A., Ashayerizadeh A., Jazi V. (2019). Synergistic effects of fermented soybean meal and mannan-oligosaccharide on growth performance, digestive functions, and hepatic gene expression in broiler chickens. Poult. Sci..

[48] Ross A. (2014). Ross Broiler Management Handbook.

[49] Amouei H., Qotbi A., Bouyeh M., Seidavi A. (2015). Effect of different levels of thyme essential oil and thechnomos prebiotic on some blood metabolites of broilers. Int. J. Biol. Pharm. Allied Sci..

[50] El-Ghousein S.S., Al-Beitawi N.A. (2009). The Effect of Feeding of Crushed Thyme (*Thymus valgaris* L) on Growth, Blood Constituents, Gastrointestinal Tract and Carcass Characteristics of Broiler Chickens. J. Poult. Sci..

[51] Cross D.E., Svoboda K., Mcdevitt R.M., Acamovic T. (2003). The performance of chickens fed diets with and without thyme oil and enzymes. Br. Poult. Sci..

[52] Placha I., Takacova J., Ryzner M., Cobanova K., Laukova A., Strompfova V., Venglovska K., Faix S. (2014). Effect of thyme essential oil and selenium on intestine integrity and antioxidant status of broilers. Br. Poult. Sci..

[53] Haselmeyer A., Zentek J., Chizzola R. (2015). Effects of thyme as a feed additive in broiler chickens on thymol in gut contents, blood plasma, liver and muscle. J. Sci. Food Agric..

[54] Alipour F., Hassanabadi A., Golian A., Nassiri-Moghaddam H. (2015). Effect of plant extracts derived from thyme on male broiler performance. Poult. Sci..

[55] Amouei H., Qotbi A., Bouyeh M., Seidavi A. (2015). Thymus vulgaris and TechnoMOS decrease Eschericia coli and increase lactobacillus in broiler ileum and cecum. Int. J. Biol. Pharm. Allied Sci..

[56] Attia Y.A., Bakhashwain A.A., Bertu N.K. (2017). Thyme oil (*Thyme vulgaris* L.) as a natural growth promoter for broiler chickens reared under hot climate. Ital. J. Anim. Sci..

[57] Attia Y.A., Bakhashwain A.A., Bertu N.K. (2018). Utilisation of thyme powder (*Thyme vulgaris* L.) as a growth promoter alternative to antibiotics for broiler chickens raised in a hot climate. Eur. Poult. Sci..

[58] Placha I., Ocelova V., Chizzola R., Battelli G., Gai F., Bacova K., Faix S. (2019). Effect of thymol on the broiler chicken antioxidative defence system after sustained dietary thyme oil application. Br. Poult. Sci..

[59] Khafar K.R., Mojtahedin A., Rastegar N., Neytali M.K., Olfati A. (2020). Dietary inclusion of thyme essential oil alleviative effects of heat stress on growth performance and immune system of broiler chicks. Iran. J. Appl. Anim. Sci..

[60] Sigolo S., Milis C., Dousti M., Jahandideh E., Jalali A., Mirzaei N., Rasouli B., Seidavi A., Gallo A., Ferronato G. (2021). Effects of different plant extracts at various dietary levels on growth performance, carcass traits, blood serum parameters, immune response and ileal microflora of Ross broiler chickens. Ital. J. Anim. Sci..

[61] Abudabos A.M., Al-Batshan H.A., Murshed M.A. (2015). Effects of prebiotics and probiotics on the performance and bacterial colonization of broiler chickens. S. Afr. J. Anim. Sci..

[62] Murshed M.A., Abudabos A.M. (2015). Effects of the dietary inclusion of a probiotic, a prebiotic or their combinations on the growth performance of broiler chickens. Rev. Bras. Cienc. Avic..

[63] Omerovic I., Milosevic B., Ciric S., Spasic Z., Lalic N., Samardzic S. (2016). Effect of prebiotic on performance and slaughter traits of broiler chickens fed lower protein diets. J. Livest. Sci..

[64] Iji P.A., Saki A., Tivey D.R. (2001). Body and intestinal growth of broiler chicks on a commercial starter diet. 2. Development and characteristics of intestinal enzymes. Br. Poult. Sci..

[65] Jiménez-Moreno E., González-Alvarado J.M., González-Serrano A., Lázaro R., Mateos G.G. (2009). Effect of dietary fiber and fat on performance and digestive traits of broilers from one to twenty-one days of age. Poult. Sci..

[66] Sojoudi M.R., Dadashbeiki M., Bouyeh M. (2012). Effect of Different Levels of Prebiotics TechnoMos on Carcass. J. Basic. Appl. Sci. Res..

[67] Sojoudi M.R., Dadashbeiki M., Bouyeh M. Effects of Different Levels of Symbiotic, Technomos on Broilers Performance. https://www.cabdirect.org/cabdirect/abstract/20133085645.

[68] Zhu X., Liu W., Yuan S., Chen H. (2014). The effect of different dietary levels of thyme essential oil on serum biochemical indices in mahua broiler chickens. Ital. J. Anim. Sci..

[69] Wade M.R., Manwar S.J., Kuralkar S.V., Waghmare S.P., Ingle V.C. (2018). Effect of thyme essential oil on performance of broiler chicken. J. Entomol. Zool. Stud..

[70] Oakley B.B., Buhr R.J., Ritz C.W., Kiepper B.H., Berrang M.E., Seal B.S., Cox N.A. (2014). Successional changes in the chicken cecal microbiome during 42 days of growth are independent of organic acid feed additives. BMC Vet. Res..

[71] Kogut M.H., Arsenault R.J. (2016). Editorial: Gut health: The new paradigm in food animal production. Front. Vet. Sci..

[72] Oakley B.B., Lillehoj H.S., Kogut M.H., Kim W.K., Maurer J.J., Pedroso A., Lee M.D., Collett S.R., Johnson T.J., Cox N.A. (2014). The chicken gastrointestinal microbiome. FEMS Microbiol. Lett..

[73] Ballou A.L., Ali R.A., Mendoza M.A., Ellis J.C., Hassan H.M., Croom W.J., Koci M.D. (2016). Development of the chick microbiome: How early exposure influences future microbial diversity. Front. Vet. Sci..

[74] Ding J., Dai R., Yang L., He C., Xu K., Liu S., Zhao W., Xiao L., Luo L., Zhang Y. (2017). Inheritance and establishment of gut microbiota in chickens. Front. Microbiol..

[75] Pan D., Yu Z. (2013). Intestinal microbiome of poultry and its interaction with host and diet. Gut Microbes.

[76] Oakley B.B., Kogut M.H. (2016). Spatial and temporal changes in the broiler chicken cecal and fecal microbiomes and correlations of bacterial taxa with cytokine gene expression. Front. Vet. Sci..

[77] Kakebaveh M., Taheri H.R., Harki Nejad T. (2014). Effects of Dietary Supplementation with Probiotics, Prebiotic and Synbiotic on Performance and Intestinal Microbial Population of Broiler Chickens. Res. Anim. Prod. Vol..

[78] Sadeghi A.A., Mohammadi A., Shawrang P., Aminafshar M. (2013). Immune responses to dietary inclusion of prebiotic-based mannan-oligosaccharide and β-glucan in broiler chicks challenged with Salmonella enteritidis. Turkish J. Vet. Anim. Sci..

[79] Al-Baadani H.H., Abudabos A.M., Al-Mufarrej S.I., Alzawqari M. (2016). Effects of dietary inclusion of probiotics, prebiotics and synbiotics on intestinal histological changes in challenged broiler chickens. S. Afr. J. Anim. Sci..

[80] Salehimanesh A., Mohammadi M., Roostaei-Ali Mehr M. (2016). Effect of dietary probiotic, prebiotic and synbiotic supplementation on performance, immune responses, intestinal morphology and bacterial populations in broilers. J. Anim. Physiol. Anim. Nutr..

[81] De Souza L.F.A., Araújo D.N., Stefani L.M., Giometti I.C., Cruz-Polycarpo V.C., Polycarpo G., Burbarelli M.F. (2018). Probiotics on performance, intestinal morphology and carcass characteristics of broiler chickens raised with lower or higher environmental challenge. Austral. J. Vet. Sci..

[82] Tayeri V., Seidavi A., Asadpour L., Phillips C.J.C. (2018). A comparison of the effects of antibiotics, probiotics, synbiotics and prebiotics on the performance and carcass characteristics of broilers. Vet. Res. Commun..

[83] Ahmadian A., Seidavi A., Phillips C.J.C. (2020). Growth, carcass composition, haematology and immunity of broilers supplemented with sumac berries (*Rhus coriaria* L.) and thyme (*Thymus vulgaris*). Animals.

